# Is Culture Expansion Necessary in Autologous Mesenchymal Stromal Cell Therapy to Obtain Superior Results in the Management of Knee Osteoarthritis?—Meta-Analysis of Randomized Controlled Trials

**DOI:** 10.3390/bioengineering8120220

**Published:** 2021-12-16

**Authors:** Sathish Muthu, Randhi Rama Kartheek, Naveen Jeyaraman, Ramya Lakshmi Rajendran, Manish Khanna, Madhan Jeyaraman, Rathinavelpandian Perunchezhian Packkyarathinam, Prakash Gangadaran, Byeong-Cheol Ahn

**Affiliations:** 1 Department of Orthopaedics, Government Medical College and Hospital, Dindigul 624001, Tamil Nadu, India; drsathishmuthu@gmail.com; 2 Department of Biotechnology, School of Engineering and Technology, Sharda University, Greater Noida 201310, Uttar Pradesh, India; 3 Indian Stem Cell Study Group (ISCSG) Association, Lucknow 226010, Uttar Pradesh, India; dr.ramkarthik@gmail.com (R.R.K.); naveenjeyaraman@yahoo.com (N.J.); manishvenus@rediffmail.com (M.K.); 4 Fellow in Orthopaedic Rheumatology, Dr. RML National Law University, Lucknow 226010, Uttar Pradesh, India; 5 Department of Orthopaedics, Atlas Hospitals, Tiruchirappalli 620002, Tamil Nadu, India; 6 Department of Nuclear Medicine, School of Medicine, Kyungpook National University, Kyungpook National University Hospital, Daegu 41944, Korea; ramyag@knu.ac.kr; 7 Department of Orthopaedics, Prasad Institute of Medical Sciences, Lucknow 226401, Uttar Pradesh, India; 8 Department of Orthopaedics, Faculty of Medicine—Sri Lalithambigai Medical College and Hospital, Dr. MGR Educational and Research Institute, Chennai 600095, Tamil Nadu, India; 9 Department of Orthopaedics, Government Medical College, Omandurar Government Estate, Chennai 600002, Tamil Nadu, India; 10 BK21 FOUR KNU Convergence Educational Program of Biomedical Sciences for Creative Future Talents, Department of Biomedical Sciences, School of Medicine, Kyungpook National University, Daegu 41944, Korea

**Keywords:** mesenchymal stromal cell, culture, bone-marrow derived mesenchymal stromal cell, adipose-derived mesenchymal stromal cell, cartilage regeneration, knee osteoarthritis, meta-analysis

## Abstract

**Study Design:** Meta-analysis. **Objectives:** We aimed to analyze the impact of cultured expansion of autologous mesenchymal stromal cells (MSCs) in the management of osteoarthritis of the knee from randomized controlled trials (RCTs) available in the literature. **Materials and Methods:** We conducted independent and duplicate electronic database searches including PubMed, Embase, Web of Science, and Cochrane Library until August 2021 for RCTs analyzing the efficacy and safety of culture-expanded compared to non-cultured autologous MSCs in the management of knee osteoarthritis. The Visual Analog Score (VAS) for pain, Western Ontario McMaster University’s Osteoarthritis Index (WOMAC), Lysholm score, Knee Osteoarthritis Outcome Score (KOOS), and adverse events were the analyzed outcomes. Analysis was performed in R-platform using OpenMeta [Analyst] software. **Results:** Overall, 17 studies involving 767 patients were included for analysis. None of the studies made a direct comparison of the culture expanded and non-cultured MSCs, hence we pooled the results of all the included studies of non-cultured and cultured types of MSC sources and made a comparative analysis of the outcomes. At six months, culture expanded MSCs showed significantly better improvement (*p* < 0.001) in VAS outcome. Uncultured MSCs, on the other hand, demonstrated significant VAS improvement in the long term (12 months) in VAS (*p* < 0.001), WOMAC (*p* = 0.025), KOOS score (*p* = 0.016) where cultured-expanded MSCs failed to demonstrate a significant change. Culturing of MSCs did not significantly increase the complications noted (*p* = 0.485). On sub-group analysis, adipose-derived uncultured MSCs outperformed culture-expanded MSCs at both short term (six months) and long term (12 months) in functional outcome parameters such as WOMAC (*p* < 0.001, *p* = 0.025), Lysholm (*p* < 0.006), and KOOS (*p* < 0.003) scores, respectively, compared to their controls. **Conclusions:** We identified a void in literature evaluating the impact of culture expansion of MSCs for use in knee osteoarthritis. Our indirect analysis of literature showed that culture expansion of autologous MSCs is not a necessary factor to obtain superior results in the management of knee osteoarthritis. Moreover, while using uncultured autologous MSCs, we recommend MSCs of adipose origin to obtain superior functional outcomes. However, we urge future trials of sufficient quality to validate our findings to arrive at a consensus on the need for culture expansion of MSCs for use in cellular therapy of knee osteoarthritis.

## 1. Introduction

The ongoing debate among global regenerative experts on cartilage regeneration is on the usage of non-cultured vs. cultured mesenchymal stromal cells (MSCs) in osteoarthritis of the knee. With the virtue of its self-renewal, multi-differentiation, and immune regulation, MSCs have been proven as promising cellular agents for cartilage regeneration. Among all the available sources of MSCs, numerous researchers paid attention to MSCs from bone marrow, adipose tissue, synovium, umbilical cord, and Wharton’s jelly. The application of MSCs through intra-articular injection or arthroscopic implantation in knee osteoarthritis appears to be safe without any major side effects [1]. Preclinical and clinical studies have demonstrated differences in the clinical outcome due to heterogeneity of cellular mixture in MSC cocktails. The influence of the total number of MSCs delivered in the inflammatory joint environment leads to suboptimal chondrogenic differentiation or rapid apoptosis of the transplanted cells. The heterogeneity of cellular mixture poses an obstacle in translational research of MSCs in cartilage regeneration in clinical practice. 

To evade the differences in the functional outcome, homogeneous cellular mixtures have to be used in optimal quantities for regenerating desired cartilaginous tissues. The major limitation of MSC therapy is the lack of standardization of the quantity of MSCs isolated from the source to meet the desired therapeutic effect (DTE). Various studies have quantified the minimum number of MSCs to produce a DTE to around 2 × 10^6^ cells per kg body weight [2,3]. However, the dose and frequency of MSCs needed may vary according to the severity of the disease [4,5]. To meet the high dose of MSCs needed for DTE, they have been culture-expanded in vitro using a cell culture plate and flask. However, the large-scale expansion of MSCs affects the quality of culture-expanded cells which may have limited potency and stemness by storing for a long period [6]. Jung et al. stated that the higher the passage number the lesser capacity of proliferation and differentiation in MSCs [7]. The culture expanded MSCs undergo spontaneous differentiation due to the presence of a heterogeneous MSC population in the cellular mixture. The addition of growth factors to such a cocktail will inhibit the spontaneous differentiation of MSCs. However, the characteristics of culture expanded MSCs, including the expression of cell surface markers and cellular viability, must be checked before application to the patients.

Various preclinical and clinical studies have demonstrated the use of culture expanded MSCs for cartilage regeneration. Centeno et al. suggested that MSCs cultured ex vivo from bone marrow aspiration of the iliac crest resulted in increased thickness of articular cartilage and regeneration of meniscus in a patient with knee osteoarthritis and meniscal injury [8]. Spakova et al. demonstrated cartilage regeneration with culturing osteochondral cylinders and bone marrow-derived MSCs from knee osteoarthritis patients along with kartogenin as a chondrogenic promoter [9]. Zhang et al. regenerated cartilage by co-culturing human Wharton jelly-derived MSCs along with primary articular cartilage cells in double biomimetic acellular cartilage extracellular matrix (ECM) scaffold in a caprine model [10]. At two years follow up, Schmal et al. regenerated the cartilage with culture-expanded allogenic synovial MSCs seeded in a collagen membrane in an osteochondral defect model of the medial femoral condyle in a rabbit [11]. The usage of cultured MSCs help in the maintenance of cartilage phenotype and improved activity of locally available primary articular chondrocytes and provides a stable chondrogenic differentiation [12]. The co-culture of MSCs along with chondrocytes provide a synergistic in vitro activation of MSCs by paracrine signaling and functional in vivo tissue regeneration [13].

With this promising evidence in literature favoring culture-expanded MSC therapy, we aim to investigate whether the use of culture-expanded autologous MSCs gives superior results compared to non-cultured autologous MSCs in the management of osteoarthritis of the knee.

## 2. Materials and Methods

We conducted this meta-analysis as per the guidelines from the Back Review Group of Cochrane Collaboration [14], and we followed the reporting guidelines of the Preferred Reporting Items for Systematic Reviews and Meta-Analyses (PRISMA) statement [15].

### 2.1. Search Strategy

**Figure 1:**
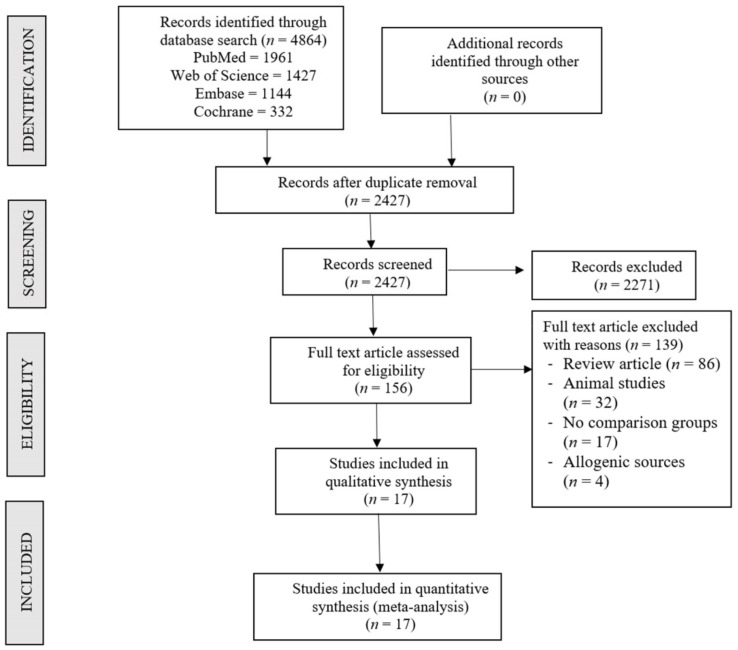
PRISMA flow diagram of the included studies.

Two reviewers performed an independent electronic literature search for studies evaluating the safety and efficacy of scaffold-based delivery of MSCs in the management of osteoarthritis of the knee. We searched the following databases: PubMed, Embase, Web of Science, and the Cochrane Library up to August 2021. No language or date restrictions were applied. Keywords used for the search were as follows: “Knee Osteoarthritis”, “Knee Degeneration”, “Stem Cell Therapy”, “Mesenchymal Stromal Cells”, “Bone marrow”, “Adipose”, “Culture-expanded”, and “Culture”. A sample search strategy used in one of the included databases is presented in the Supplementary Materials (Table S1). The reference list of the selected articles was also searched to identify studies not identified in the primary search. As per the inclusion and exclusion criteria, eligible studies were included for meta-analysis. The discrepancy between the authors was resolved through discussion until a consensus was obtained. A detailed study selection flow diagram is given in Figure 1.

### 2.2. Inclusion Criteria

Studies were included for quantitative review if they met the following PICOS criteria:
**Population:**Patients with knee osteoarthritis.**Intervention:**Culture-expanded MSC therapy.**Comparator:**Non-cultured MSC therapy.**Outcomes:**Visual Analog Score (VAS) for Pain, Western Ontario McMaster Universities Osteoarthritis Index (WOMAC), Lysholm Knee Scale (Lysholm), Knee Osteoarthritis Outcome Score (KOOS), and adverse events.**Study Design:**Randomized Controlled Trials.

### 2.3. Exclusion Criteria

Trials were excluded if they had the following characteristics:

1. RCTs on MSC based therapy for knee osteoarthritis without mention on the source of MSCs utilized in the study;
2. In vitro studies involving stem cell therapy;
3. Studies of observational nature and interventional studies without an appropriate comparison group;
4. Studies conduction animal models of knee osteoarthritis investigating stem cell therapy;
5. Review articles and in vitro studies involving stem cell therapy.

### 2.4. Data Extraction

Two reviewers retrieved independently relevant data from articles included for analysis. Following data were extracted:

1. Study characteristics: year of publication, authors, country, level of evidence, number of patients enrolled;
2. Baseline characteristics: mean age, gender proportions, Kellgren Lawrence grade of osteoarthritis, source of MSC utilized, intervention for both the groups, delivery method of MSCs, follow-up duration, and assessment parameters utilized;
3. Efficacy Outcomes: VAS for pain, Functional outcomes like WOMAC score, Lysholm and KOOS score;
4. Safety Outcomes: Adverse events in the included studies.

Any disagreement in data collection was resolved until a consensus was attained by discussion.

### 2.5. Risk of Bias and Quality Assessment

The methodological quality of the included studies was assessed independently by two reviewers using The Cochrane Collaboration’s ROB2 tool for randomized studies, which has five domains of bias assessment including randomization process, deviation from intended intervention, missing outcome data, measurement of the outcome, and selection of the reported results [16].

### 2.6. Statistical Analysis

Meta-analysis was conducted in the R platform with OpenMeta [Analyst] [17]. For dichotomous variable outcomes, a risk ratio (RR) with 95% Confidence Interval (CI) was used, and for continuous variable outcomes, a weighted mean difference (WMD) with 95% CI was used. Heterogeneity was assessed using the I^2^ test [18]. If I^2^ < 50% and *p* >0.1, we used a fixed-effects model to evaluate, otherwise, a random-effects was used. A *p*-value < 0.05 was considered significant. Sensitivity analyses were performed to explore the source of heterogeneity when it existed. Subgroup analysis was performed when heterogeneity was noted in the results analyzed from the included studies. Publication bias was analyzed with a funnel plot and normal quantile plot for the outcomes in the included studies and Egger’s regression test.

## 3. Results

### 3.1. Search Results

An electronic database search resulted in 4864 articles which, after initial screening for duplicate removal, gave a total of 2427 articles. Title and abstract screening were performed in those 2427 articles, and 2271 of them were excluded. 156 articles qualified for full-text review, of which 139 were excluded. None of the studies screened made a direct comparison between the culture expanded MSCs and non-cultured MSCs in the management of knee osteoarthritis. Hence, we pooled the results of all the included studies utilizing autologous culture expanded MSCs into one group and non-cultured MSCs into another group, and performed a combined comparative quantitative analysis. We included 17 included studies [19,20,21,22,23,24,25,26,27,28,29,30,31,32,33,34,35] with 767 patients for meta-analysis. A PRISMA flow diagram of study selection is given in Figure 1. Overall, 9/17 studies [21,22,24,25,26,27,28,30,35] utilized culture expanded MSCs, of which three studies used adipose-derived MSCs [21,25,30], and the remaining six studies utilized MSCs of bone marrow origin. Overall, 8/17 studies [19,20,23,29,31,32,33,34] utilized non-cultured MSCs, of which four studies utilized MSCs of adipose origin [23,29,31,32,33], three studies utilized MSCs of bone marrow origin [19,20,34], and one study [19] used both bone marrow and adipose tissue as their MSC source. All of the studies using culture expanded MSCs used cells before the fourth passage. There was also no uniformity among the included studies for the outcome measures utilized. The general characteristics of the studies included were given in Table 1. The protocol of intervention used in the case and control groups along with the measures of outcome assessment were given in Table 2.

### 3.2. Quality Assessment

**Figure 2:**
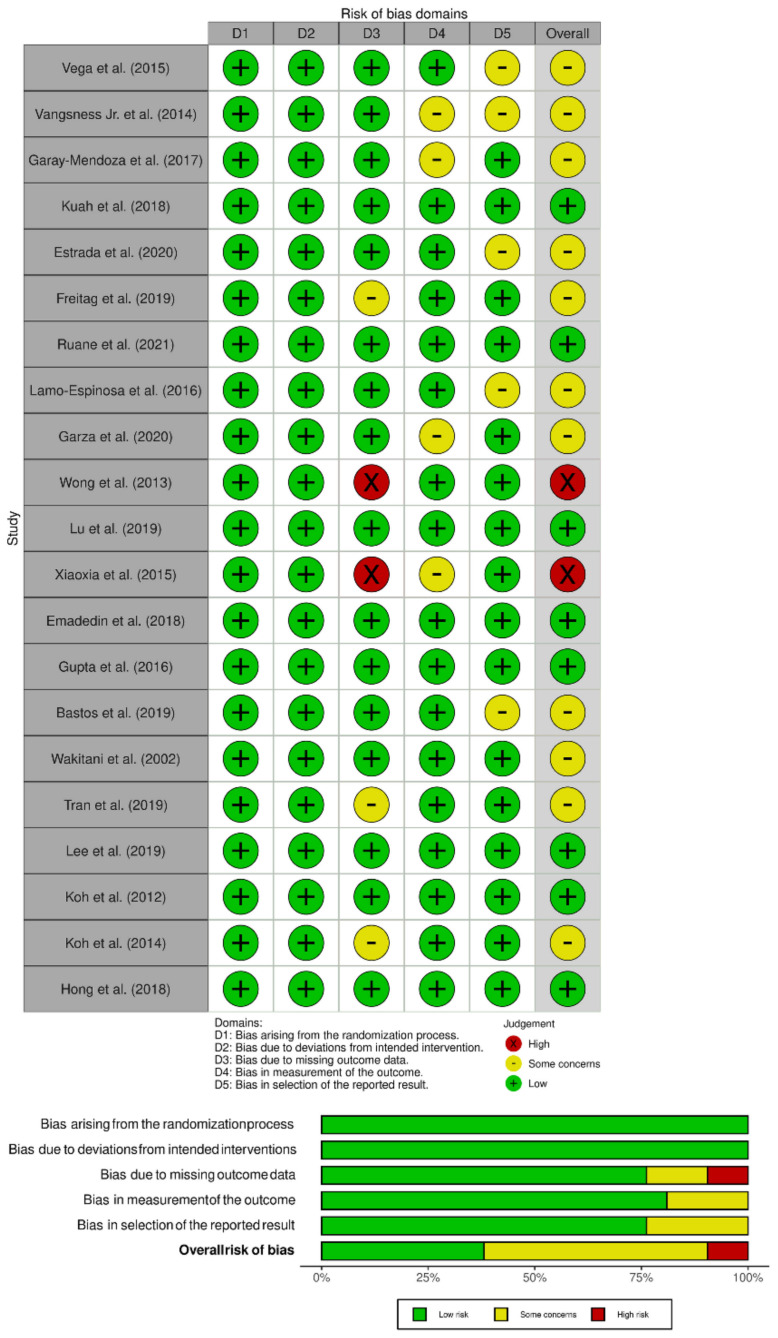
Methodological quality and risk of bias assessment of all the included studies.

The methodological quality of the included studies evaluated as per the RoB2 tool is presented in Figure 2. None of the included have has risk arising from the randomization process or due to deviation from the intended intervention, but we noted some concerns in 4/17 studies [20,23,35,36] regarding the measurement of outcome measure due to the variability and non-availability of outcome data across all the timepoints of follow-up of the patients. Similarly, 5/17 studies [19,22,27,36,37] also had some concerns with the selective reporting of the results observed due to the selective reporting of significant results across the various timepoints of follow-up of the patients. Overall 3/17 studies [21,29,31] and 2/17 studies [24,35] had some concerns and high risk with respect to the missing outcome data, which did not prevent them from being included in the analysis, since they had outcome data at the final follow-up.

### 3.3. Efficacy Outcomes

#### 3.3.1. Visual Analog Scale for Pain

**Figure 3:**
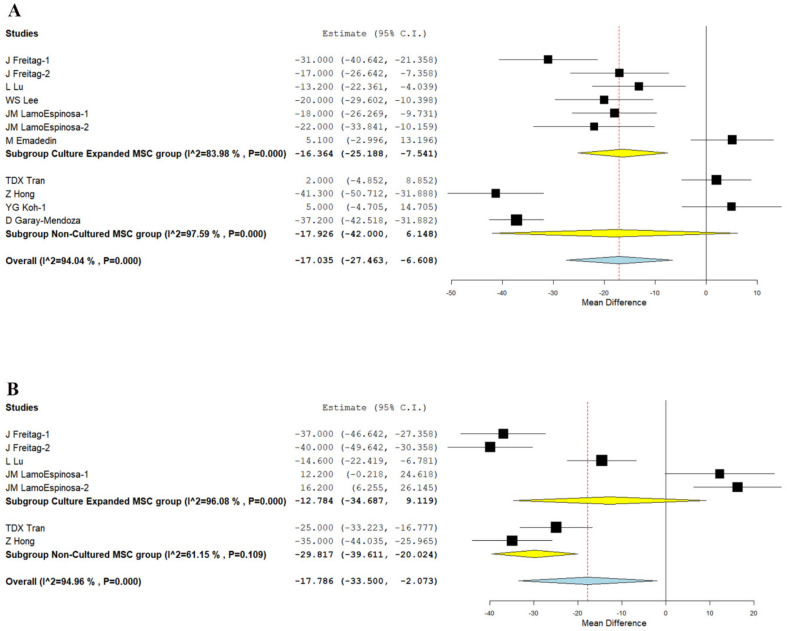
Forest plot of the included studies comparing culture-expanded and non-cultured MSCs. (**A**): VAS at 6 months showing a significant reduction in studies using culture-expanded MSCs (WMD = −16.364, *p* < 0.001) whereas studies utilizing uncultured MSCs did not produce a significant change compared to their controls (WMD = −17.926, *p* = 0.144); (**B**): VAS at 12 months showing a significant reduction in studies using uncultured MSCs (WMD = −29.817, *p* < 0.001), whereas studies using culture-expanded MSCs did not produce a significant change compared to their controls (WMD = −12.784, *p* = 0.253). Bold text gives a summation of the subgroup analyzed.

We analyzed five studies [21,22,25,26,30], and four studies [20,29,31,33] reporting the VAS outcome of culture-expanded and non-cultured MSCs, respectively, at six months. There was a significant heterogeneity observed between the included studies. (I^2^ > 80%, *p* < 0.001). Hence, the random-effects model was used for analysis. On analysis, a significant reduction in VAS score was noted compared to their controls at six months in studies using culture-expanded MSCs (WMD = −16.364, 95% CI [−25.188, −7.541], *p* < 0.001), whereas studies utilizing non-cultured MSCs did not produce a significant change in VAS scores compared to their controls (WMD = −17.926, 95% CI [−42.000, 6.148], *p* = 0.144) as shown in Figure 3A.

Similarly, we analyzed three studies [21,22,25], and two studies [29,33] reporting the VAS outcome of culture-expanded and non-cultured MSCs, respectively, at 12 months. There was a significant heterogeneity observed between the included studies. (I^2^ > 80%, *p* < 0.001). Hence, the random-effects model was used for analysis. On analysis, a significant reduction in VAS score was noted compared to their controls at 12 months in studies using non-cultured MSCs (WMD = −29.817, 95% CI [−39.611, −20.024], *p* < 0.001), whereas in studies using culture expanded MSCs (WMD = −12.784, 95% CI [−34.687, 9.119], *p* = 0.253; Figure 3B) could not produce a significant difference compared to their controls as shown in Figure 3B.

Hence, our analysis shows that studies using culture-expanded sources of MSCs were able to produce significant short-term (six months) pain relief while non-cultured MSCs produced significant pain relief in the long term (12 months).

#### 3.3.2. WOMAC Score

**Figure 4:**
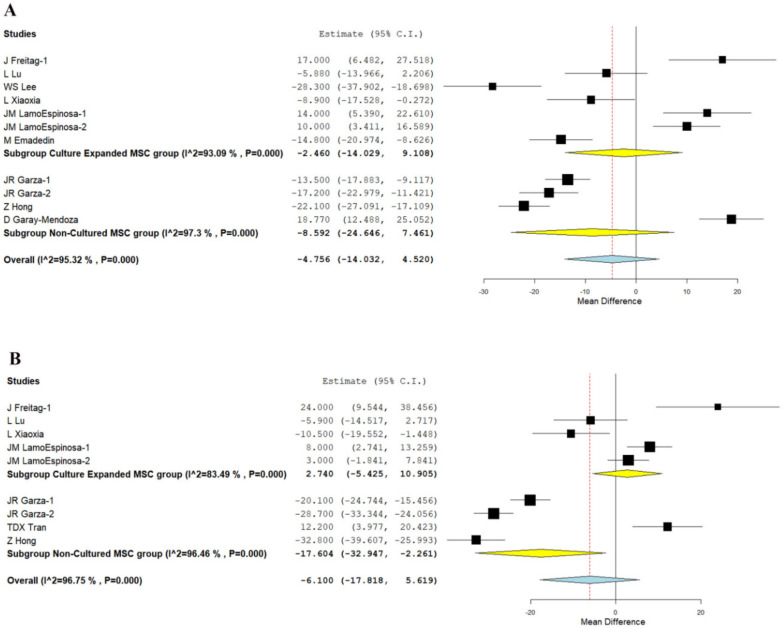
Forest plot of the included studies culture-expanded and non-cultured MSC therapy for knee osteoarthritis compared to their controls. (**A**): WOMAC at six months; B: WOMAC at 12 months. (**A**): At six months, neither studies using culture expanded MSCs (WMD = −2.460, *p* = 0.677) nor uncultured MSCs (WMD = −8.592, *p* = 0.294) demonstrated any significant change in WOMAC score compared to their controls; (**B**): WOMAC at 12 months showing a significant reduction in studies using uncultured MSCs (WMD = −17.604, *p* = 0.025), whereas studies using culture-expanded MSCs did not produce a significant change compared to their controls (WMD = 2.740, *p* = 0.511). Bold text gives a summation of the subgroup analyzed.

We analyzed six studies [21,22,25,26,30,35], and three studies [20,23,33] reporting the WOMAC scores of using culture-expanded and non-cultured sources of MSCs, respectively at six months. There was a significant heterogeneity observed between the included studies. (I^2^ > 80%, *p* < 0.001). Hence, the random-effects model was used for analysis. On analysis, significant improvement in WOMAC score was not noted in either culture-expanded group (WMD = −2.460, 95% CI [−14.029, 9.108], *p* = 0.677) compared to their controls at six months, or non-cultured sources of MSCs (WMD = −8.592, 95% CI [−24.646, 7.461], *p* = 0.294) compared to their controls, as shown in Figure 4A.

Similarly, we analyzed four studies [21,22,25,35], and three studies [23,29,33] reporting the WOMAC scores of using culture-expanded and non-cultured sources of MSCs, respectively, at 12 months. There was a significant heterogeneity observed between the included studies. (I^2^ > 80%, *p* < 0.001). Hence, the random-effects model was used for analysis. On analysis, a significant reduction in WOMAC score was noted compared to their controls at 12 months in studies utilizing non-cultured MSCs (WMD = −17.604, 95% CI [−32.947, −2.261], *p* = 0.025), whereas culture expanded MSCs (WMD = 2.740, 95% CI [−5.425, 10.905], *p* = 0.511) could not produce a significant difference compared to their controls as shown in Figure 4B.

On analysis of the WOMAC score reduction potential of both types of MSCs, it is noted, as shown in Figure 4, that most of the studies that utilized culture expanded MSCs did not report any significant improvement compared to their controls. On the other hand, although studies that utilized non-cultured MSCs did not demonstrate improvement in WOMAC scores in short term (six months), they produced a significant change in the long term (12 months) compared to their controls. Since the WOMAC score concentrates more on the functional efficiency of the intervention apart from pain reduction, non-cultured MSCs stand superior to culture expanded MSCs to provide better functional results in long term.

#### 3.3.3. KOOS Score

**Figure 5:**
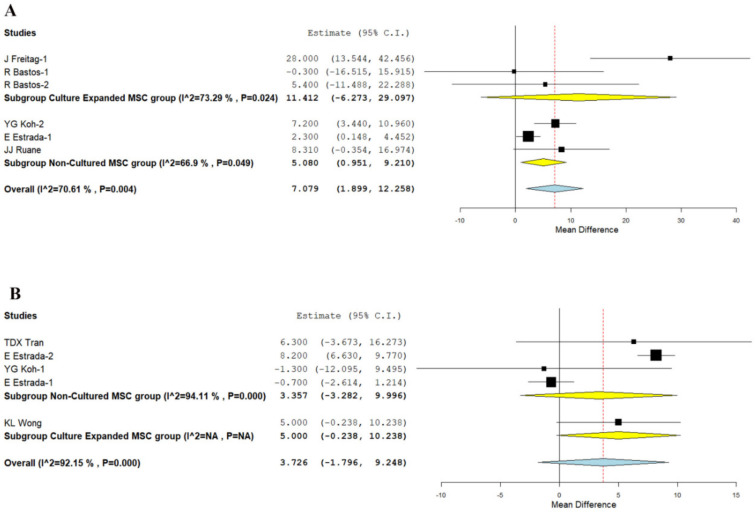
Forest plot of the included studies comparing culture-expanded and non-cultured MSC therapy for knee osteoarthritis compared to their controls. (**A**): KOOS score at 12 months; (**B**): Lysholm at 12 months. (**A**): KOOS score at 12 months showing a significant improvement in studies using uncultured MSCs (WMD = 5.080, *p* = 0.016), whereas studies using culture-expanded MSCs did not produce a significant change compared to their controls (WMD = 11.412, *p* = 0.206); (**B**): At 12 months, neither studies using culture-expanded MSCs (WMD = 5.000, *p* = 0.054) nor uncultured MSCs (WMD = 3.357, *p* = 0.322) demonstrated any significant change in Lysholm score compared to their controls. Bold text gives a summation of the subgroup analyzed.

We analyzed two studies [21,27], and three studies [19,32,34] reporting the KOOS score at 12 months using culture expanded and non-cultured MSCs, respectively. There was a significant heterogeneity observed between the included studies. (I^2^ = 70.61%, *p* = 0.004). Hence, the random-effects model was used for analysis across all time points. On analysis, a significant improvement in KOOS score was noted compared to their controls at 12 months in studies utilizing non-cultured MSCs (WMD = 5.080, 95% CI [0.951, 9.210], *p* = 0.016), whereas culture expanded MSCs (WMD = 11.412, 95% CI [−6.273, 29.097], *p* = 0.206) could not produce a significant difference compared to their controls as shown in Figure 5A.

On critical analysis of the improvement in the KOOS score in the non-cultured MSCs group compared to the culture-expanded group, it is noted that only in the non-cultured group was significant improvement in the functional outcomes noted, which is in corroboration with the WOMAC scores of both the groups.

#### 3.3.4. Lysholm Knee Score

We analyzed three studies [19,29,31], and one study [24] reporting the Lysholm score at 12 months using culture expanded and non-cultured MSCs, respectively. There was a significant heterogeneity observed between the included studies. (I^2^ > 80%, *p* < 0.001). Hence, the random-effects model was used for analysis across all time points. On analysis, both the culture-expanded (WMD = 5.000, 95% CI [−0.238, 10.238], *p* = 0.054) and the non-cultured (WMD = 3.357, 95% CI [−3.282, 9.996], *p* = 0.322) did not produce any significant improvement in scores compared to their controls at 12 months, as shown in Figure 5B.

#### 3.3.5. Safety

**Figure 6:**
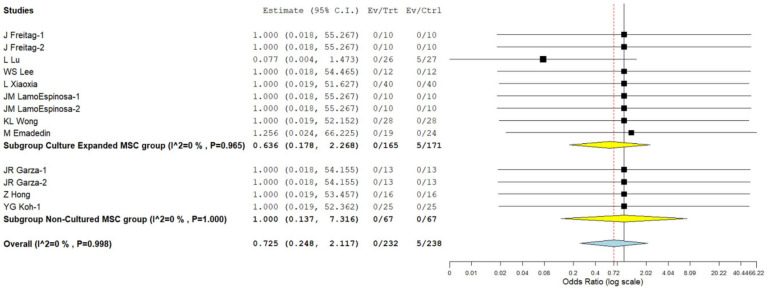
Forest plot of the included studies comparing adverse events upon culture expanded and non-cultured MSC therapy for knee osteoarthritis compared to their controls. Bold text gives a summation of the subgroup analyzed.

Overall, seven studies [21,22,24,25,26,30,35] involving 336 patients reported adverse effects with low heterogeneity among the included studies using culture-expanded MSCs for knee osteoarthritis. (I^2^ = 0.0%, *p* = 0.965). Hence, a fixed-effects model was used for analysis. On analysis, we did not note any significant increase in the adverse events compared to the controls. (OR = 0.636, 95% CI [0.178, 2.268], *p* = 0.485; Figure 6) Similarly, we analyzed three studies [23,31,33] involving 134 patients reporting adverse events with low heterogeneity among the included studies using an autologous source of MSCs for knee osteoarthritis. (I^2^ = 0.0%, *p* = 0.998). Hence, a fixed-effects model was used for analysis. There was no significant increase in the adverse events compared to the controls. (OR = 1.000, 95% CI [0.137, 7.316], *p* = 1.000; Figure 6). No major serious adverse events with permanent effects such as death, tumor, or immune reaction to the intervention were noted during follow-up in either of MSC types. Hence it is evident from the analysis that culture expanded MSCs are safer, as compared to non-cultured MSCs for knee osteoarthritis.

#### 3.3.6. Sensitivity Analysis

A sensitivity analysis was performed in each analysis. All the results (VAS for Pain, WOMAC, KOOS, Lysholm, and adverse events) were not significantly altered by sequentially omitting each study in the meta-analysis. On the other hand, the consistency of the results was maintained after reanalysis by changing to the random-effects model.

#### 3.3.7. Subgroup Analysis

We produced a subgroup analysis of all the results based on the source of MSCs, such as adipose tissue and bone marrow, to analyze their influence on the heterogeneity of the outcomes analyzed. The results of the subgroup analysis are presented in Table 3. No change in the results of the culture expanded MSCs group was noted upon sub-group analysis. However, it was evident from the analysis that, lack of significance of the results in the non-cultured group in the functional parameters, such as WOMAC and Lysholm, was contributed by the cell of origin of MSCs used in them. Hence, upon using adipose tissue as the source of non-cultured MSCs for knee osteoarthritis, consistent significant results were noted in functional parameters such as WOMAC, Lysholm, and KOOS scores at various time points analyzed without significant heterogeneity. Due to the lack of sufficient data, we could not analyze the impact of the method of culturing and number of cell passages on the outcomes observed.

#### 3.3.8. Publications Bias

**Figure 7:**
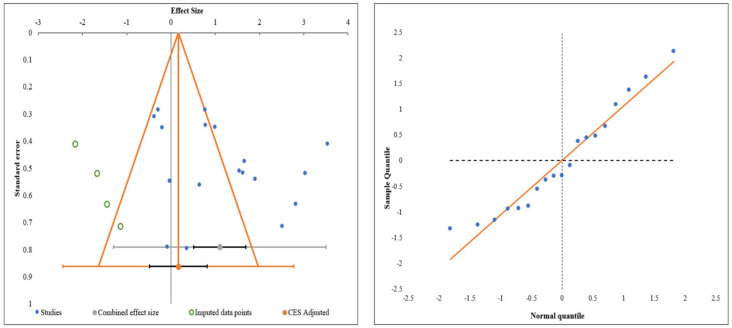
Publication bias assessment with funnel plot and quantile plot for Visual Analog Score at six months in the included studies.

Publication bias was analyzed utilizing the funnel plot, normal quantile plot, and Egger’s regression test for the meta-analysis performed. There was no evidence of publication bias by funnel plot and normal quantile plot, as shown in Figure 7, or by Egger’s regression test (*p* = 0.519). All of the studies lay close to the 95% CI, and no significant heterogeneity was noted in the distribution of the studies about the axes, implying minimal publication bias.

## 4. Discussion

The usage of MSCs in clinical practice is growing day by day, and various research organizations are generating robust evidence for various indications. Due to the versatile nature of MSC, cell biology has become the building block in translational research on tissue engineering, and regenerative medicine. The culture expansion of cells in animal model date back to the 1970s [36,37] In 1992, the first isolation and culture expansion of human bone marrow-derived MSCs were reported [38], and the re-implantation of such culture expanded cells were reported in 1995 [39] The most commonly utilized MSC sources are bone marrow and adipose tissue, whereas umbilical cord and placenta, which are considered as medical waste, are also rich in MSCs [40].

Isolation of MSCs from a source and re-implantation at the target site with DTE is still under debate. The optimal number of MSCs required to treat a tissue injury remain unanswered by regenerative biologists. To obtain DTE and excellent functional and clinical outcomes, culture expansion of MSCs have been considered with the rationale that increased cells would increase the functional results. Through cell culturing methods, 25 mL of bone marrow can produce 100 to150 million clinical-grade MSCs in approximately three to four weeks in a packed volume of about 0.4 to 0.5 mL [40,41].

Lamo-Espinosa et al. [22] performed a study in 30 symptomatic knee osteoarthritis patients with intra-articular hyaluronate for 10 patients as a control group; intra-articular hyaluronate with 10 × 10^6^ and 100 × 10^6^ cultured autologous bone marrow-derived MSCs for 10 patients in each dosage group, respectively, and followed up for 12 months. They concluded that single intra-articular injection of in vitro culture-expanded 100 × 10^6^ autologous bone marrow-derived MSCs along with hyaluronate as a safe and feasible orthobiologic procedure for symptomatic knee osteoarthritis [22]. However, the above cell count could be delivered without the need for culturing methods.

Similarly, Garza et al. [23] performed a dose escalation with a non-cultured adipose tissue-derived stromal vascular fraction (SVF) with 13 patients of the high-dose group (3 × 10^7^ SVF cells); 13 patients of the low-dose group (1.5 × 10^7^ SVF cells); and 13 patients of the placebo control group (zero SVF cells). They concluded that non-cultured intra-articular SVF significantly reduces pain irrespective of the dose, but no difference was discovered in cartilage thickness with the follow-up MRI [23]. These results were in accordance with the findings of our analysis.

### 4.1. Main Finding

We comprehensively and critically reviewed all the available literature to identify the necessity of culture-expansion in the MSC therapy for knee osteoarthritis and found that:

1. Although at six months, culture expanded MSCs showed significantly better VAS improvement (*p* < 0.001), it was not consistent at 1 year (*p* = 0.253). Non-cultured MSCs, on the other hand, demonstrated significant VAS improvement in the long term (*p* < 0.001), which was not noted in short term (*p* = 0.144).
2. Similarly, adipose-derived non-cultured MSCs outperformed culture-expanded MSCs in both the short term (six months) and long term (12 months) in functional outcome parameters, such as WOMAC (*p* < 0.001, *p* = 0.025), Lysholm (*p* < 0.006), and KOOS (*p* < 0.003) scores, respectively, compared to their controls.
3. No significant adverse events were noted in either culture expanded MSC (*p* = 0.485) or non-cultured MSC (*p* = 1.000) groups compared to their controls.

The culture expanded MSCs pose various risk benefits to the patients in the clinical practice. The ideal culture expansion must be carried out in a GMP-certified stem cell laboratory [42]. The evaluation of potential risk benefits is a mandatory step in the usage of culture expanded MSC products. The possible risks involved in using cultured MSCs depend on the nature (autologous/allogeneic) and type of stem cells used, their proliferation and differentiation status, methods employed for isolation from culture, and re-implantation to the target site of action. Other factors that play a role in the culturing methods include the number of passages, minimally or more than minimally manipulation of cells, methods utilized to ensure their safety and efficacy. Apart from the factors mentioned, concerns about the long-term survival of engrafted cells, their therapeutic benefits in terms of tissue regeneration, and anticipated complications such as activation of immune responses, the transmission of viral proteins, tumorigenicity, genetic changes, and chromosomal aberrations need to be considered, which is not the case with the uncultured MSCs, since they can be administered instantaneously upon separation in a single surgical window.

Utilization of culture-expanded MSCs poses major morbidity for patients in terms of the two-stage procedure with the first stage for isolation of cells to be culture expanded and the other for re-implantation of cultured cells in the target site of action. Moreover, the culture expansion of cells results in a heterogeneous group of cells. During isolation of MSCs with collagenase from the culture, the biological properties of MSCs may be hampered [43]. In vitro culture of MSC in an optimal hypoxic state increases the regenerative potential of cells, increases the lifespan of MSCs, and decreases oxidative stress, telomere shortening, and chromosomal aberrations [44]. However, no standardized protocols were available in many laboratories for in vitro isolation of MSCs from the tissues which greatly affects the clinical outcomes. The criteria laid by the International Society for Cellular Therapy (ISCT) for MSC characterization are sometimes not met in the cultured MSCs, which further causes discrepancies in their efficiency [45].

During the cellular passage, the proteolytic enzyme poses damage to ECM protein and modifies the inherent property of activation of intracellular signaling pathways which produce an inferior quality of the cellular product [46]. The freezing agent dimethyl sulfoxide (DMSO) causes toxicity to various cells, hence cryopreserved cultured cells must be thawed before transplantation [47]. The cellular proliferation in the culture media depends on the source of MSCs, stage of the cells, culture condition, and culture seeding density [48]. 3D culture expansion increases the therapeutic effect of traditional 2D culture expansion [49]. The MSC culture media should be free of bovine components, as they transmit prion diseases and activate the immune system of organ recipients. Instead, autologous plasma or allogenic platelet lysate enhances the MSC growth and proliferation in the culture [50,51].

Long-term culture expansion results in cellular senescence, growth arrest, and cellular apoptosis along with the reduction of therapeutic properties [52]. Few studies provide controversial evidence of the malignant transformation of cultured MSCs [53,54,55,56], whereas a few researchers have reported genetic transformation of culture-expanded MSCs following in vivo MSC transplantation [57,58]. Long term in vitro culture expansion result in higher genetic instability such as DNA damage and chromosomal aberrations in MSCs, hence it warrants regular monitoring of ex vivo culture expansion to improve the therapeutic safety and efficacy [59].

The biological tissue can be grown in different culture conditions and exposed to test the product for safety and efficacy following different in vitro culture methods [60,61,62]. Although we use more modern systems for in vitro cultures, the definition of the test system is needed to check the pliability of the biological, chemical, or physical system in the finalized platforms. Microfluidics and micro-physiological technologies are used for testing human stem cell products [63,64]. To date, no standardized method is adopted for regulatory purposes, despite the developments in technology progresses. Good Cell Culture Practice produced a set of principles to be followed for working with in vitro cell and tissue culture systems, including isolation, culture expansion, separation, manipulation, transport of cells, transplantation to the target site, and adverse effects testing and reporting [65,66,67]. Before using any cellular product, the user should check for solid ethical provenance, safety assessment, and intellectual property rights [60,68].

Tissues to be cultured must be procured from an authorized tissue retrieval bank to avoid viral contamination. In the case of no tissue banks being available, there should be an agreed testing method in place regarding all aspects of harvesting, preparation, labeling, storage, and transfer [69]. The culture expansion must not alter the biological properties of cells or tissues. The cells can only be minimally or more than minimally manipulated, and are utilized only for homologous use [70]. Biological materials fall under “Dangerous Goods” during shipping, and must comply with International Air Transport Association (IATA) regulations and Dangerous Goods Regulations (DGR) [71,72,73].

It is always recommended in case of utilizing allogenic culture-expanded MSCs in large-scale commercial products to follow a two-tiered cell banking system consisting of a Master Cell Bank (MCB) and a Working Cell Bank (WCB) [69,74,75]. MCB consists of 10 to 20 vials of 1 mL each containing 1–5 × 10^6^ cells, and is not for distribution and must be protected from unintended use. From one MCB, WCBs should be created when required. A single vial from MCB is thawed and cultured to create the WCB. WCBs must contain sufficient ampoules of cells to be utilized for the proposed experimental procedure or task in a defined period. When working with biological tissues of an animal or human origin, it is mandatory to follow the national guidelines and legislation [69]. Culture expanded MSC products have been developed by various research groups and were tested clinically for the product’s safety and efficacy. CARTISTEM, an allogenic culture-expanded umbilical cord blood-derived MSCs, was used at a dose of 2.5 × 10^6^ cells/500 μL/cm^2^ area of knee cartilage for the cartilage defect [76]. Gupta et al. used an allogenic product named STEMPEUCELL, ex vivo culture-expanded pooled allogeneic human bone marrow-derived MSCs, at a dose of 2 × 10^8^ cells cryopreserved and stored in 15 mL cryo-bags for cartilage regeneration in symptomatic knee osteoarthritis [77]. These products, CARTISTEM and STEMPEUCELL, have been developed for carrying out investigational research in the field of regenerative medicine. However, future clinical trials of large scale might be warranted to recommend their routine clinical usage. Although we have various restrictions and guidelines governing the implementation and practice of culture-expanded cell products, the current literature does not support culture expansion as a necessary essential method to obtain superior functional benefits in MSC-based therapy for knee osteoarthritis.

### 4.2. Limitations

Our analysis has some limitations. Blinding was not established in most of the studies, which might invite room for treatment bias from patients or observers. Heterogeneity was observed in most of the outcomes reported across the studies, which might be due to the variability in the treatment protocols followed in the individual studies as shown in Table 2, which was not explored in the subgroup analysis. Moreover, patients in various stages of the disease process were included in the studies, which might also contribute to the heterogeneity of their results. We recommend a large multicentric trial analyzing the need for culture expansion of MSCs with standardized dosage and intervention protocols, evaluated with established outcome measures both in the short and long term, without any adjuvant procedures to further confirm the results of our analysis.

## 5. Conclusions

We identified a void in literature evaluating the impact of culture expansion of MSCs for use in knee osteoarthritis. Our indirect analysis of literature showed that culture expansion of autologous MSCs is not a necessary factor to obtain superior results in the management of knee osteoarthritis. Moreover, while using uncultured autologous MSCs, we recommend MSCs of adipose origin to obtain superior functional outcomes. However, we urge future trials of sufficient quality to validate our findings to arrive at a consensus on the need for culture expansion of MSCs for use in cellular therapy of knee osteoarthritis.

## Figures and Tables

**Table 1:** Characteristics of included studies.

Sl. No	Study	Year	Country	Nature of Study	Kellgren Lawrence Grade	Sample Size	Treatment/ Control	Mean Age (SD)		Male/Female		MSC Type	Culture Expanded/ Non-Cultured	Follow-Up (Months)
								Treatment Group	Control Group	Treatment Group	Control Group			
1	Garay-Mendoza et al.	2017	Mexico	RCT	NR	61	30/31	55.57 ± 12.02	59.32 ± 10.85	07/23	09/22	BM	NC	6
2	Estrada et al.	2020	Argentina	RCT	I, II, III	89	60/29	61 ± 12	61 ± 12	NR	NR	BM/AD	NC	12
3	Freitag et al.	2019	Australia	RCT	II, III	30	20/10	54.6 ± 6.3	51.5 ± 6.1	11/09	01/09	AD	CE	12
4	Ruane et al.	2021	USA	RCT	I, II, III	32	17/15	58.06 ± 9.14	58.6 ± 8.05	09/08	10/05	BM	NC	12
5	Lamo-Espinosa et al.	2016	Spain	RCT	II, III, IV	30	20/10	65.9	60.3	12/08	07/03	BM	CE	12
6	Garza et al.	2020	USA	RCT	II, III	39	26/13	60.5 ± 7.9	57.1 ± 9.1	15/11	7/6	AD	NC	12
7	Wong et al.	2013	Singapore	RCT	NR	56	28/28	53	49	15/13	14/14	BM	CE	24
8	Lu et al.	2019	China	RCT	I, II, III	53	27/26	55.03 ± 9.19	59.64 ± 5.97	03/24	03/23	AD	CE	12
9	Xiaoxia et al.	2015	China	RCT	I, II	80	40/40	55.9 ± 8.1	55.1 ± 6.8	14/26	13/27	BM	CE	12
10	Emadedin et al.	2018	Iran	RCT	II, III, IV	43	19/24	51.7 ± 9.2	54.7 ± 5.3	12/07	15/09	BM	CE	6
11	Bastos et al.	2019	Brazil	RCT	I, II, III, IV	47	30/17	55.7 ± 7.8	55.9 ± 13.4	15/15	09/08	BM	CE	12
12	Wakitani et al.	2002	Japan		I, II	24	12/12	NR	NR	NR	NR	BM	CE	16
13	Tran et al.	2019	Taiwan	RCT	II, III	33	15/18	58.2 ± 5.70	59.0 ± 6.04	03/12	05/13	AD	NC	24
14	Lee et al.	2019	South Korea	RCT	II, III, IV	24	12/12	62.2 ± 6.5	63.2 ± 4.2	03/09	03/09	AD	CE	6
15	Koh et al.	2012	South Korea	RCT	IV	50	25/25	54.2 ± 9.3	54.4 ± 11.3	08/17	08/17	AD	NC	16
16	Koh et al.	2014	South Korea	RCT	I, II, III	44	23/21	52.3 ± 4.9	54.2 ± 2.9	06/17	05/16	AD	NC	24
17	Hong et al.	2018	China	RCT	II, III	32	16/16	51 ± 5.95	53 ± 10.97	03/13	03/13	AD	NC	12

AD—Adipose derived; Allo—Allogenic; Auto—Autologous; BM—Bone Marrow derived; CE—Culture Expanded; MSC —Mesenchymal Stem Cell; NC—Non-Cultured; NR—Not Reported; RCT—Randomized Controlled Trial; SD—Standard Deviation; USA—United States of America.

**Table 2:** Stem cell transplantation protocol of the included studies.

Study	MSC Type	MSC Source	MSC Preparation	MSC Count (10^7^ cells)	Treatment Group Intervention	Control Group Intervention	Outcome Measures
Garay-Mendoza et al.	BM	Auto	BMC	NA	600 µg/day G-CSF for 3 consecutive days before the procedure + sIA Injection of MSC	Oral acetaminophen 500 mg every 8 h for 6 months	VAS, WOMAC
Estrada et al.	AD	Auto	BMC	NA	sIA Injection of bone marrow concentrate	sIA Injection of PRP	IKDC, Lysholm Score, KOOS
Estrada et al.	BM	Auto	SVF	NA	sIA Injection of lipoaspirate	sIA Injection of PRP	
Freitag et al.	AD	Auto	CE- ADMSC	10	sIA Injection of MSC ± 2nd injection at 6 months	Conservative management	VAS, WOMAC, KOOS, MRI assessment
Ruane et al.	BM	Auto	BMC	NA	sIA Injection of bone marrow concentrate + PRP	Gel-One^®^ Cross-Linked Hyaluronate injection	VAS, KOOS
Lamo-Espinosa et al.	BM	Auto	CE-BMMSC	1	sIA Injection of MSC +60 mg HA	sIA Injection of 60 mg HA	VAS, WOMAC, MRI assessment
Garza et al.	AD	Auto	SVF	NA	sIA Injection of MSC	Placebo injection without cells	WOMAC, MRI assessment
Wong et al.	BM	Auto	CE-BMMSC	1.46	HTO + Microfracture + sIA Injection of MSC + 20 mg HA	HTO + Microfracture + sIA Injection of 20 mg HA	Tegner Score, Lysholm Score
Lu et al.	AD	Auto	CE- ADMSC	5	2 IA Injection of MSC at 0, 3 weeks and sham injection at 1, 2 weeks	4 IA Injection of 25 mg HA at 0, 1, 2, 3 weeks	VAS, WOMAC
Xiaoxia et al.	BM	Auto	CE-BMMSC	3.82	3 × Monthly IA Injection of MSC + 20 mg HA	sIA Injection of 20 mg HA	Tegner Score, Lysholm Score
Emadedin et al.	BM	Auto	CE-BMMSC	4	sIA Injection of MSC	Placebo sIA Injection of Normal Saline	VAS, WOMAC
Bastos et al.	BM	Auto	CE-BMMSC	4	sIA Injection of MSC in 10 mL of PRP	sIA Injection of 4 mg Dexamethasone	KOOS, MRI assessment
Wakitani et al.	BM	Auto	CE-BMMSC	1	HTO + Microfracture + sIA Injection of MSC	HTO + Microfracture + Placebo injection	MRI assessment, HSS Knee rating scale
Tran et al.	AD	Auto	SVF	NA	Arthroscopic micro fracture + sIA Injection of MSC	Arthroscopic micro fracture	WOMAC, MRI assessment
Lee et al.	AD	Auto	CE- ADMSC	10	sIA Injection of MSC	Placebo injection with Normal Saline	WOMAC, MRI assessment
Koh et al.	AD	Auto	SVF	0.189	Arthroscopic debridement + sIA Injection of MSC + PRP	Arthroscopic debridement + PRP	VAS, Tegner Score, Lysholm Score
Koh et al.	AD	Auto	CE- ADMSC	0.411	HTO + sIA Injection of MSC + PRP	HTO + PRP	VAS, Lysholm Score
Hong et al.	AD	Auto	SVF	0.745	sIA Injection of MSC	sIA Injection of 40 mg HA	VAS, WOMAC, MRI assessment

AD—Adipose derived; Allo—Allogenic; Auto—Autologous; BM—Bone Marrow derived; BMC—Bone Marrow Concentrate; CE-ADMSC—Culture Expanded Adipose Derived MSC; CE-BMMSC—Culture Expanded Bone Marrow MSC; HA—Hyaluronic Acid; HSS—Hospital for Special Surgeries; HTO—High Tibial Osteotomy; IA—Intra-articular; IKDC—International Knee Documentation Committee, KOOS—Knee Osteoarthritis Outcome Score; PRP—Platelet Rich Plasma; MRI—Magnetic Resonance Imaging; MSC—Mesenchymal Stem Cells; sIA—Single Intra-articular; SVF—Stromal Vascular Fraction; VAS—Visual Analog Score; WOMAC—Western Ontario Mc-Master Universities Osteoarthritis Index.

**Table 3:** Subgroup analysis exploring into the heterogeneity of the results.

Outcomes	Culture Expanded MSCs						Uncultured MSCs					
	Bone Marrow Derived MSCs			Adipose Derived MSCs			Bone Marrow Derived MSCs			Adipose Derived MSCs		
	Estimate	95% CI	*p*-Value	Estimate	95% CI	*p*-Value	Estimate	95% CI	*p*-Value	Estimate	95% CI	*p*-Value
VAS—6 months	−11.344	−28.555, 5.867	0.196	−21.319	−31.512, −11.125	**<0.001**	−37.200	−42.518, −31.882	NA	−11.366	−39.218,16.487	0.424
VAS—12 months	14.637	6.875, 22.399	**<0.001**	−30.328	−47.004, −13.652	**<0.001**	NA	NA	NA	−29.817	−39.611, −20.024	**<0.001**
WOMAC—6 months	5.139	−7.847, 18.124	0.438	5.303	−17.114, 27.719	0.643	18.770	12.488, 25.052	NA	−17.508	−22.715, −12.302	**<0.001**
WOMAC—12 months	0.967	−7.659, 9.594	0.826	8.464	−20.815, 37.742	0.571	NA	NA	NA	−17.604	−32.947, −2.261	**0.025**
Lysholm Score—12 months	5.000	−0.238, 10.238	NA	NA	NA	NA	−0.700	−2.614, 1.214	NA	6.494	1.889, 11.100	**0.006**
KOOS Score—12 months	2.434	−9.262, 14.130	0.683	28.000	13.544, 42.456	NA	3.780	−1.295, 8.854	0.144	5.083	1.729, 8.437	**0.003**
Adverse Events	1.047	0.177, 6.200	0.960	0.377	0.061, 2.327	0.293	NA	NA	NA	NA	NA	NA

CI—Confidence Interval; KOOS—Knee Osteoarthritis Outcome Score; MSC—Mesenchymal Stem Cells; NA—Not Applicable; VAS—Visual Analog Score; WMD—Weighted Mean Difference; WOMAC—Western Ontario Mc-Master Universities Osteoarthritis Index. Significant difference in the outcome measures analyzed were presented in bold numbers.

## Data Availability

Data will be shared upon request.

## Supplementary Materials

The following are available online at https://www.mdpi.com/article/10.3390/bioengineering8120220/s1, Table S1: Search strategy used in PubMed database.
