# Supplementary material for: Is Culture Expansion Necessary in Autologous Mesenchymal Stromal Cell Therapy to Obtain Superior Results in the Management of Knee Osteoarthritis?—Meta-Analysis of Randomized Controlled Trials
Source: Bioengineering (Basel). 2021 Dec 16;8(12):220. doi: 10.3390/bioengineering8120220 (PMC8698637; doi:10.3390/bioengineering8120220)
Supplement: Supplementary file 1 [file bioengineering-08-00220-s001.zip › bioengineering-1486763-supplementary.pdf]

bioengineering

MDPI

Supplementary Materials

Table S1. Search strategy used in PubMed database.

Search Topic	#	Search Terms
Population: *Human subjects with knee osteoarthritis*	#1	("Osteoarthritis"[Mesh]) OR (osteoarthritis [All Fields]) OR (OA[All Fields])
	#2	("knee joint"[MeSH]) OR ("knee joint"[All Fields]) OR ("knee"[All Fields])
	#3	#1 AND #2
Intervention: *Cellular therapy, Injection, Culture Expanded BM-MSC, AD-MSC, Bone marrow, Adipose tissue*	#4	("stem cell transplantation"[Mesh]) OR ("cell- and tissue-based therapy"[Mesh]) OR ("mesenchymal stem cells"[Mesh]) OR ("culture expanded"[All Fileds]) OR ("stem cell therapy"[All Fields]) OR ("cellular therapy"[All Fields]) OR ("stem cell therapy"[All Fields]) OR ("mesenchymal stem cell therapy"[All Fields])
	#5	("bone marrow"[Mesh]) OR ("adipsoe tissue"[Mesh]) OR ("bone marrow transplantation"[Mesh]) OR ("bone marrow"[All Fields]) OR ("adipsoe tissue"[All Fields]) OR ("bone marrow transplantation"[All Fields])
	#6	#4 AND #5
	#7	#3 AND #6
Study design: *RCTs*	#8	("clinical trial"[All Fields] OR "clinical trials as topic"[MeSH Terms] OR "clinical trials"[All Fields] OR “"randomized controlled trial"[All Fields] OR "randomized controlled trials as topic"[MeSH Terms] OR "randomized controlled trial"[pt] OR "controlled clinical trial"[pt] OR "randomized"[tiab] OR "randomly"[tiab] OR "trial"[ti] OR "randomised controlled trial"[All Fields] OR "randomized controlled trial"[pt] "prospective studies"[MeSH Terms] OR "prospective studies"[All Fields]
	#9	#7 AND #8
	#10	("retrospective studies"[MeSH Terms] OR "retrospective studies"[All Fields] OR "retrospective study"[All Fields] OR “biography”[Publication Type] OR “comment”[Publication Type] OR “directory”[Publication Type] OR “editorial”[Publication Type] OR “festschrift”[Publication Type] OR “interview”[Publication Type] OR “lecture”[Publication Type] OR “legal case”[Publication Type] OR “legislation”[Publication Type] OR “letter”[Publication Type] OR “news”[Publication Type] OR “newspaper article”[Publication Type] OR “patient education handout”[Publication Type] OR “popular work”[Publication Type] OR “congress”[Publication Type] OR “consensus development conference”[Publication Type] OR “consensus development conference, nih”[Publication Type] OR “practice guideline”[Publication Type]) OR “Review”[Publication Type])
	#11	#9 NOT #10
